# Elucidation of Mechanism of Action in Drug Invention: Using Stable Isotope Tracers to Unravel Biochemical Kinetics

**DOI:** 10.1002/prp2.70099

**Published:** 2025-04-25

**Authors:** Rebecca A. Kohnz, Dan Zhou, Bin Lou, Huifang Yao, David McKenney, Dhiraj Dokwal, Ruth Villanueva, Heidi Kocalis, Jeanine E. Ballard, Jennifer Piesvaux, Stephen F. Previs

**Affiliations:** ^1^ Merck & co., Inc. South San Francisco California USA; ^2^ Merck & co., Inc. West Point Pennsylvania USA; ^3^ Merck & co., Inc. Rahway New Jersey USA; ^4^ Merck & co., Inc. Boston Massachusetts USA

**Keywords:** biochemical pathway, drug discovery, isotope tracers, mass spectrometry, metabolic activity, organ crosstalk, pharmacology

## Abstract

The invention of a therapeutic begins by characterizing features that differentiate healthy versus diseased states; this often presents as changes in the concentration of an analyte. Examples include elevated blood glucose in diabetes, high cholesterol in heart disease, and protein aggregation in neurodegeneration. Analyte concentrations reflect the (im)balance of synthetic and degradation rates; as such, aberrant biochemical kinetics drive the changes in endpoint concentration that define disease biology. Therapeutics aim to reset the concentration of a disease marker via modulation of biochemical kinetics. This is easy to understand for drugs directly targeting an enzyme in a pathway but, although less obvious, this can also be at the core of protein: protein interactions. For instance, stimulation of the insulin receptor changes the flux of several biochemical substrates (across multiple tissues); similarly, modulation of proprotein convertase subtilisin/kexin type 9‐low density lipoprotein (PCSK9‐LDL) receptor interactions alters cholesterol trafficking. These classic examples underscore the importance of studying biochemical kinetics at a clinical level. Here, we discuss how kinetic studies link disease biology with mechanism of action elucidation and screening. This has an immediate impact on (i) enabling in vitro*‐*in vivo correlations in early discovery, (ii) enhancing exposure‐response models aiding in human dose prediction, and (iii) providing support for biomarker plans, including clinical diagnostics. Mechanism of action studies can also influence modality selection; e.g., knowledge regarding target kinetics is needed when making decisions surrounding the development of a reversible inhibitor vs. an irreversible covalent modifier, or an intervention that affects target levels such as those which enhance protein degradation or reduce protein synthesis.

## Graphical abstract

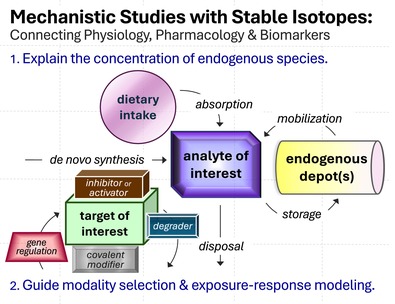

## 1 Introduction to the Importance of Biochemical Kinetics in Disease Pathophysiology

**FIGURE 1:**
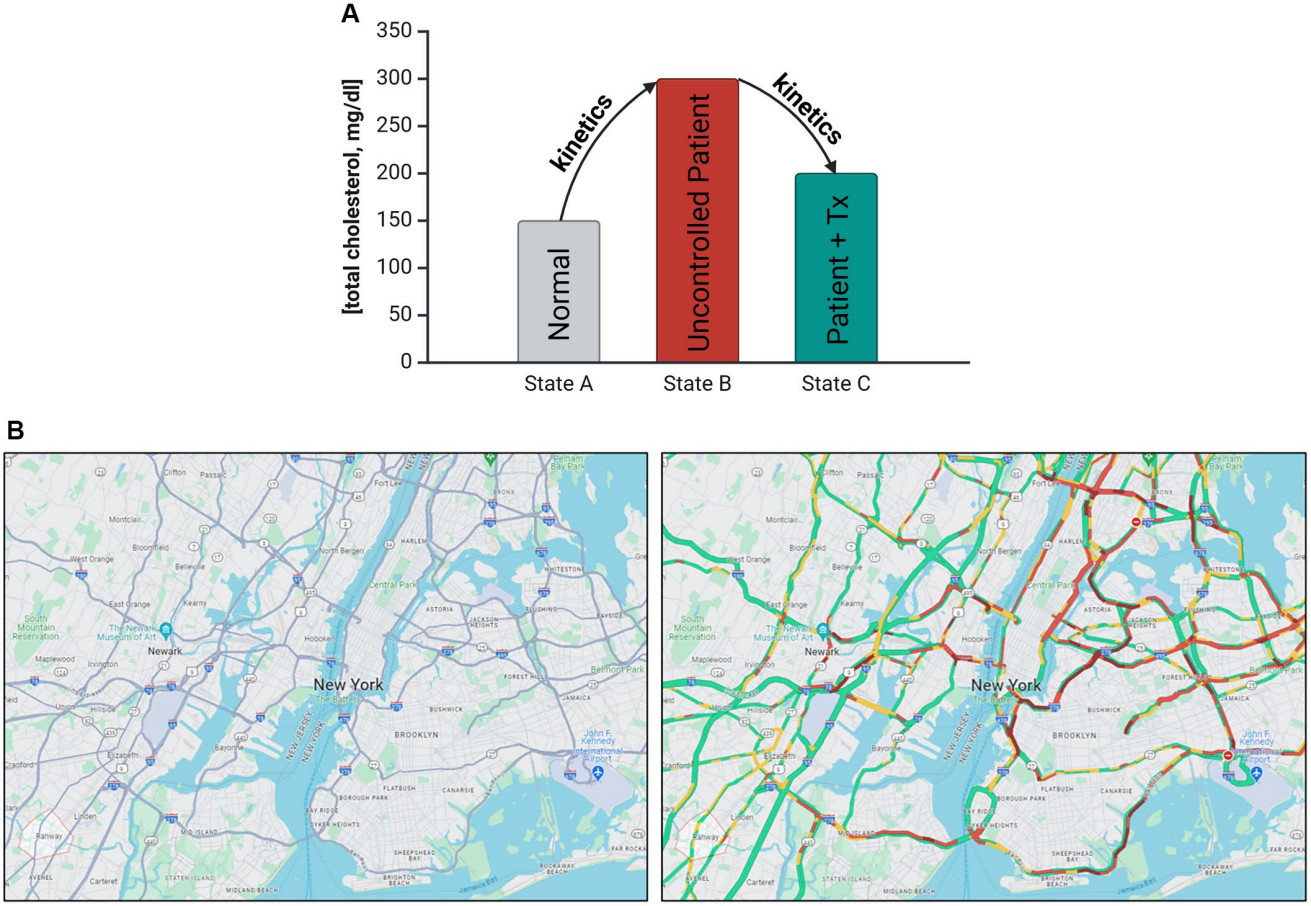
Differentiation of health status. The concentration of various analytes (e.g., substrates, intermediates and/or products) is often used to determine the presence or absence of disease; likewise, therapeutics aim to restore the concentration of respective analytes to normal values (Panel A) [1]. Analyte concentrations reflect the balance between inputs and outputs in a system, e.g., production and disposal. Stable isotope tracers can be used to estimate rates of production and/or disposal of a given analyte (Panel A). MS‐based assays can yield measures of analyte concentration, as such they can be used to build roadmaps that define sizes of various pools (Panel B); when stable isotope tracers are coupled with MS assays it is possible to assign patterns of biochemical flow like a driver utilizing a traffic overlay (download from maps.google.com).

Integrative physiology and organ crosstalk are at the core of many diseases, as altered metabolic control leads to changes in the levels of various analytes that typically differentiate “normal” from “abnormal” biology (Figure 1A). Several assay formats can be used in clinical and basic research to define health status; for example, in some cases genomics will yield insight regarding the etiology of disease (e.g., newborn screening for phenylketonuria) whereas in other cases a defined diagnostic challenge (e.g., a glucose tolerance test) shines light on the potential for transitioning between “good” and “bad” states. Whereas the latter scenario has a more immediate link between aberrant kinetics and illness—namely, the body is (un)able to dispose of a known dose of substrate in a given timeframe—newborn screening methods rely on static measures of metabolite concentrations to connect alterations with genetic defects that impact metabolic regulation and control pathway kinetics.

Whether kinetics are directly or indirectly evaluated, e.g., glucose tolerance testing or screening for inborn errors of metabolism, respectively, the importance of metabolic trafficking in medicine is clear. Since isotope tracer methods are ideally suited for inferring movement, it is not surprising that various methods are used to guide patient classification and/or to follow the progression/regression of disease. For example, “Fluorodeoxyglucose‐Positron Emission Tomography” (FDG‐PET) is widely used to locate malignant areas of high glucose metabolism in oncology [2]. This important kinetic method also yields a whole‐body measure of the spatial distribution of cancerous cells. Comparable stable isotope probes and assays are being developed [3, 4], including hyperpolarized substrates [5], to further enhance knowledge of local metabolic activity. These examples require that a subject travel to a specialized center with the requisite instrumentation, however, isotope tracer methods can be applied in a flexible environment (e.g., home or standard outpatient office) and the collected samples are sent to an analytical site. For example, subjects can be given an oral dose of ^13^C‐urea, after which breath samples can be collected and shipped to a laboratory to measure ^13^C‐labeled CO_2_, enabling an assessment of the presence of *Helicobactor pylori* [6]. Likewise, novel protocols can be used to examine hepatic flux rates in outpatient‐type settings [7].

Clinically, isotope‐based kinetic tests are available to support diagnoses and guide the effectiveness of interventions. In this review, we will focus on the broader utility of isotope labeling studies in the development of therapeutics and connect the reader from bench to bedside. The discussion is biased towards the use of stable isotopes, as they offer a safer alternative as compared to radioactive probes and can be coupled with various detection systems (e.g., mass‐ or nuclear magnetic resonance spectrometers, MS and NMR, respectively) to yield specificity of labeling in a given analyte [8]. A deep comparison of methodology is not considered here, but it is useful to note that MS methods are often faster and more sensitive as compared to NMR, though NMR yields novel insight in studies that require detailed knowledge of positional labeling. Whereas NMR has long been used to study spatial metabolism yielding real‐time kinetic images [9, 10], recent advances in MS offer the promise of comparable readouts in some instances [11, 12].

## 2 Using Stable Isotopes to Study Biochemical Kinetics

While numerous assay formats can determine the concentration of a given analyte, we take examples from our experience at Merck & Co. Inc., Rahway, NJ, USA, in which stable isotope tracer methods are coupled with MS readouts to delve mechanistically into target biology. A simple analogy is drawn from using maps when driving to get from Point A to B. For example, we can use a map to identify city size, differentiate highways from country roads, and estimate mileage; these static measures suggest what is possible when getting from Point A to B (Figure 1B). Similarly, MS assays yield a measure of size (or amount) of an analyte regardless of its associated biological system (e.g., the same assay can be used to measure glucose whether it comes from a cell or preclinical in vivo model or a patient); these measures provide a list of possibilities for characterizing a phenotype. However, if a driver needs to get from Point A to B quickly, it is also necessary to understand the current traffic patterns; coupling MS assays with isotope tracers allows researchers to identify the route that contributes to an altered analyte level (Figure 1B) and therein ascribe the mechanism of action (MoA).

### 2.1 Intersectionality of Biochemical Kinetics and the Drug Discovery Workflow

**FIGURE 2:**
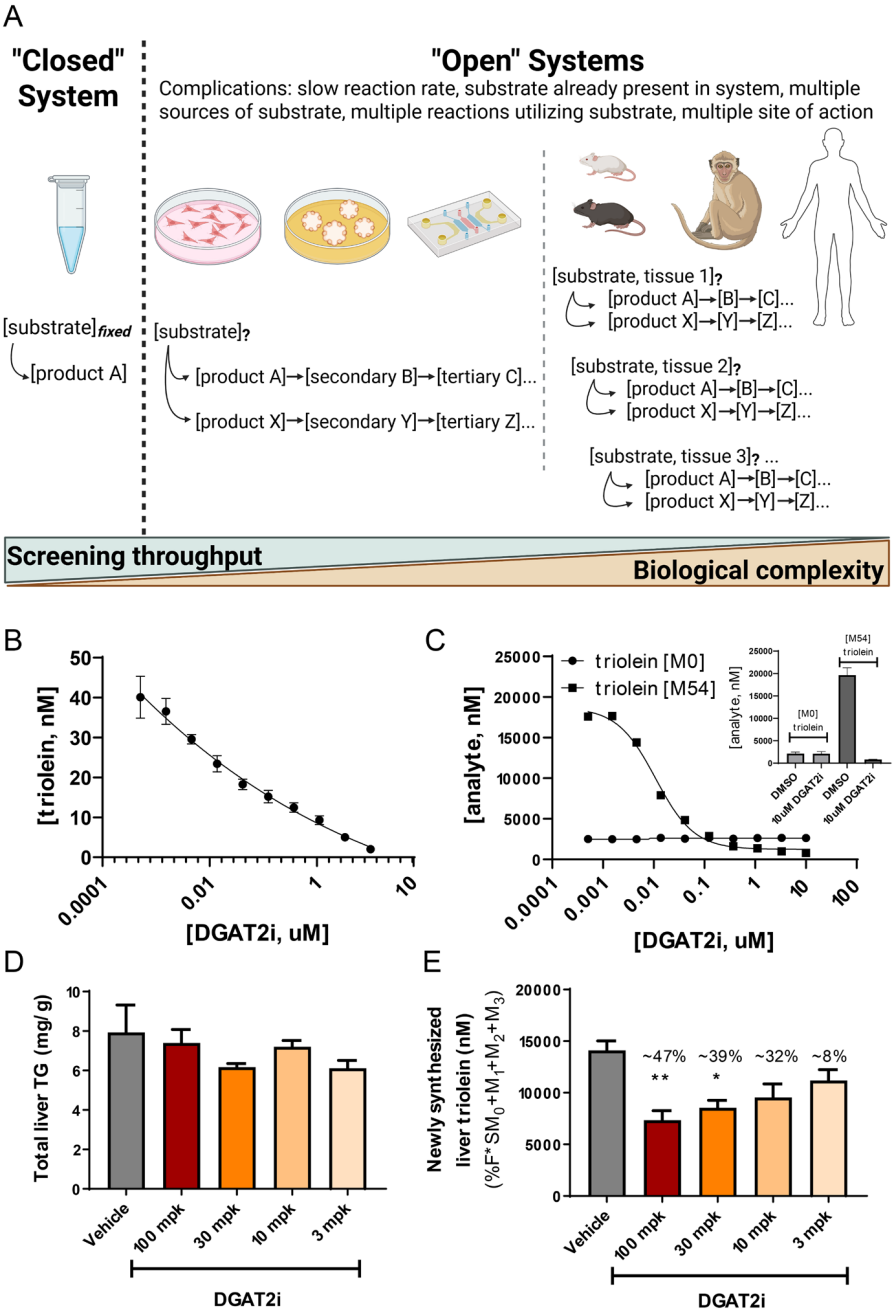
Evolving complexity in the discovery workflow. The use of target‐based screening during the invention of classical small molecule drugs follows the general path contained in Panel A [13]. The activity of a purified (e.g., recombinant) protein is typically measured in a “closed” system, allowing tight control of the conditions. Molecules of interest move down the workflow into “open” systems wherein reaction schemes become more complex, metabolic activity against the substrate of interest can be influenced by many additional factors. Our initial efforts to generate an inhibitor of DGAT2 yielded a compound of interest, referred to as DGAT2i herein (Panel B). Without utilization of a stable isotope precursor, an “open” cell‐based assay would have had a signal:background of nearly zero response (Panel C, inset, compare endogenous triolein “M0” to newly made fully labeled ^13^C‐triolein “M54”). A dose–response curve would have been impossible to generate without utilization of the ^13^C_18_‐oleate precursor in a cellular assay (Panel C). Furthermore, this same compound in vivo demonstrated virtually no immediate (acute) impact on triglyceride levels (Panel D). When the compound was dosed in parallel with a ^13^C‐fatty acid tracer, we observed a reasonable exposure‐response (Panel E). A caveat here is that a single triglyceride species is being measured, studies determined that this one species does reflect changes in the overall pool.

Our logic can be considered in the context of drug invention. In a typical target‐based screen, early assays in the hit‐finding funnel typically rely first on “closed” systems; one tests the ability of compounds to alter a reaction rate in a test tube with tightly controlled variables (e.g., defined buffer composition, a known amount of target protein, and a fixed substrate concentration) (Figure 2A). MS is well suited for this early discovery work, especially when sensitivity and specificity are crucial for successful screening.

Compounds with sufficient potency in a “closed” system move down the funnel and undergo further evaluation in more complex “open” systems such as cell‐based and in vivo models (Figure 2A). Unfortunately, weaker apparent potency is commonly observed when molecules are tested in “open” models. Chemists and pharmacologists recognize that dosing a known amount of compound in “open” systems requires knowledge beyond a half‐maximal effective concentration (EC_50_), primarily that of absorption‐distribution‐metabolism‐elimination (ADME) parameters which determine true exposures. Cellular biochemistry presents an analogous problem on metabolic regulation (Figure 2A). Readouts in “open” systems can be confounded since (i) the reaction of interest may be slower, (ii) the substrate and/or product may already be present, (iii) a single target may have multiple substrates/products, (iv) there may be more than one pathway or site of action to create a product, (v) many substrates/products are not dead‐end molecules, themselves being subject to additional biochemical reactions [14]. While not an exhaustive list, these variables make it such that measurements of analyte concentrations may lead to errors when estimating potency and/or efficacy in “open” biochemical networks [15].

Within the drug development workflow, coupling MS assays with stable isotope tracers can illuminate the gray area that arises when pharmacology attempts to modulate target biology. One notable example comes from our diacylglycerol O‐acyltransferase 2 (DGAT2) program. DGAT2 catalyzes the esterification of diacylglycerol and long‐chain fatty acyl‐CoAs, which is the final reaction in triacylglycerol (triglyceride, TG) synthesis. As DGAT2 plays a critical role in the generation of TGs and high levels of TG are associated with several negative clinical outcomes, e.g., insulin resistance, hyperlipidemia, and fatty liver disease, the rationale was to block TG synthesis via pharmacologic modulation (lowering) of DGAT2 activity. Although medicinal chemistry efforts successfully generated DGAT2 small molecule inhibitors as assessed by a biochemical assay (Figure 2B) [16], it was quickly noted that compounds with good potency in biochemical assays had a limited effect on cellular TG (Figure 2C, inset, “M0” signal represents endogenous triolein). Potency concordance was only observable when the cellular assay was adapted to use a stable isotope precursor, [U‐^13^C_18_]oleate, to probe the DGAT2‐catalyzed formation of “M54” [U‐^13^C_18_]‐triolein (Figure 2C, inset, “M54” signal represents newly made triolein). In this case, the tracer‐based readout clearly demonstrated that the compound in cells exerted an expected response (Figure 2B,C) on a newly synthesized TG, namely the triolein species. Similarly, no response in acute in vivo models was seen when measuring the concentration of total hepatic TGs (Figure 2D). In this case, the administration of the same isotopically labeled fatty acid precursor ([U‐^13^C_18_]oleate) similarly demonstrated an unambiguous outcome on pathway activity when DGAT2 activity was inhibited (Figure 2E).

Reflecting on biochemical kinetics, it is important to recognize that the tracer allowed quantification of TG synthesis via movement of new molecules into the total TG pool, whereas measuring a bulk response (lowering of the total TG level) would require more time than the acute in vivo target engagement study could offer. We direct an interested reader to a more complete discussion of the technical nuance in the literature [16, 17]; this example underscores the importance of understanding endogenous turnover. The time it takes to observe changes in total pool size (bulk response) is related to the half‐life of the endpoint of interest and the delta one aims to observe. For example, after one half‐life a researcher expects the concentration will decrease by 50%; however, that assumes complete suppression of the input. To extend our example, the early DGAT2 drug candidates being studied are unlikely to have optimized potency and/or pharmacokinetic properties. Under these conditions, a study letting one half‐life elapse may not result in the expected 50% decrease in product pool since inhibition of TG synthesis may not be 100%. It is important to note that drug studies do not always require flux readouts; researchers can run a tracer experiment ahead of drug screening to determine the system kinetics and from there establish an optimal time for sampling bulk responses. If the tracer pilot study informs on a relatively long half‐life of the endogenous readout (or biomarker) then it may justify the use of tracers on the critical path to success for the screening and development workflow. And lastly, another important confounding issue to consider here is that one also assumes that input sources and output sinks for the endogenous readout do not change with pharmacologic intervention or during the progression of a disease model. Unfortunately, there is no guarantee that knocking down one enzyme/pathway will not provoke a counter‐regulatory response; this will limit the overall effectiveness of a treatment that aims to change the level of a biomarker. In cases where branch points exist in a pathway, isotope flux analyses can also yield insight regarding the source(s) of new product molecules.

Since flux rates are likely different across in vitro cell types, various preclinical animal models, and humans, tracer studies can generate critical knowledge of metabolic (or biochemical) kinetics for building in vitro*–*in vivo correlations, therein helping to predict or account for delays in responses as programs move from early discovery (e.g., cell models) to in vivo systems up to and including clinical trials.

Previous efforts realized the value of studying pathway activity surrounding compounds that (i) moved deep into preclinical development, e.g., adenosine 5'‐monophosphate‐activated protein kinase (AMPK) activator [18, 19], (ii) required MoA elucidation when transitioning to clinical development, e.g., novel insulins [20, 21], or (iii) yielded unexpected outcomes while in clinical development, e.g., acetyl‐CoA carboxylase (ACC) inhibitor [22], glucagon receptor antagonist [23]. Although these examples demonstrate the utility of tracer kinetics in the context of late‐stage development, there is value added by adopting this mindset in the early stages of drug discovery as a translational bridge into the clinic. For example, researchers might have little knowledge of underlying pathway activity and need to examine the potential for cross‐connectivity, e.g., fatty acid synthase (FAS) inhibition [24], including unexpected compensation, e.g., DGAT2 inhibition [16].

**FIGURE 3:**
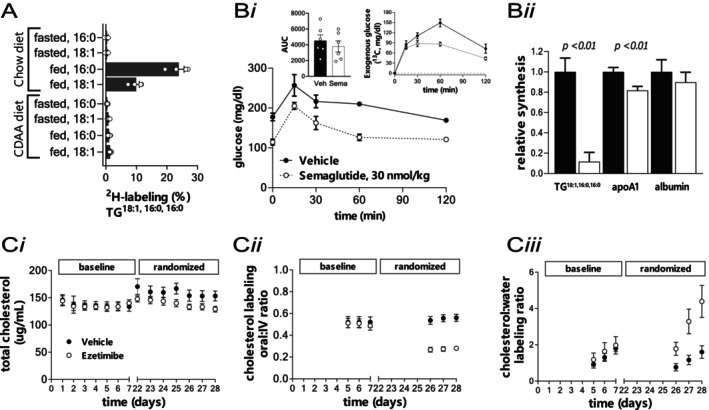
Model selection and pathway interconnectivity. Data can have various interpretations at the interface of disease area biology and in vivo pharmacology. Panel A demonstrates that a “chow” diet has a marked effect on fatty acid synthesis in mice that are either fed or fasted overnight, this is in strong contrast to the effect of feeding a choline deficient‐high fat “CDAA” diet. This information can be used to evaluate the utility of a given diet model on pathway tone. Panel B demonstrates a Semaglutide‐mediated lowering of baseline (fasting) glucose in mice made obese via high‐fat diet (Bi). Although the oral glucose tolerance test did not identify any effect on the dynamic response of glucose (no change in the stimulated AUC, Bi, left inset), since the perturbation contained ^13^C‐glucose, MS analyses demonstrated an effect on glucose entry to the plasma following treatment with Semaglutide (Bi, right inset). Subsequent testing, wherein mice were given an intraperitoneal bolus of ^2^H‐water, revealed Semaglutide‐mediated effects on selected lipid and protein synthesis (Bii). Panel C demonstrates pharmacodynamic interactions surrounding cholesterol biology. Exposure to Ezetimibe slightly reduced total cholesterol (Ci, *p* = 0.12) despite driving a nearly 50% decrease in absorption (Cii, *p* < 0.05). Additional analyses demonstrated a shift in the contribution of cholesterol synthesis (Ciii, *p* < 0.05), which presumably off‐set some of the lowering potential that could be achieved by blocking absorption.

Next, we briefly outline three scenarios where isotope methods can be incorporated in research programs to understand interactions between pharmacology and biology (Figure 3). It should be obvious that a researcher's firm control of experimental variables is quickly lost as a program moves from biochemical screening to cell models, to preclinical in vivo studies, and finally into patients. In biochemical screening, virtually every reagent can be qualified and quantified; cellular assays can also offer some degree of homogeneity (e.g., a single cell line is incubated in a known media) but add complexity since many simultaneous ongoing reactions are present. Animal models (in vivo) have some points where tight control can be held (e.g., genetic strain, use of specific diets) but again, new variables appear since one now has to contend with organ cross‐talk and integration between fed and fasted states. Finally, humans introduce a vast number of complex variables including diverse genetic backgrounds, lifestyles, age, pre‐existing conditions, etc. As these variables expand and/or change across the development path, it is reasonable to expect that pathway activity may not be conserved; we briefly review three examples where characterization of metabolic profiles can help align models, unmask hidden phenotypes, and illuminate crosstalk.

First, several preclinical models can be used to support studies aimed at modulating fatty liver disease, e.g., a choline deficient high fat (CDAA) diet leads to pronounced liver disease in mice [25]. We demonstrated that the CDAA diet model, when compared to a carbohydrate‐based “chow” diet, is associated with virtually no activity of the de novo lipogenic (DNL) pathway (Figure 3A). This represents a stark disconnect between current views on the role of DNL in the clinical etiology versus the generation of preclinical models to support pharmacology and drug discovery programs, suggesting little value in using the CDAA model to test hypotheses regarding inhibition of DNL despite the marked effect of the CDAA diet on liver health [7, 26, 27].

Second, taking a glucagon‐like peptide‐1 (GLP‐1) agonist as an example, we examined in vivo glucose tolerance during sub‐chronic dosing of Semaglutide in mice (Figure 3Bi, 30 nmol ∙ kg^−1^ ∙ day^−1^; weight loss and changes in food intake were observed, not shown). The use of an isotopically labeled glucose challenge study can differentiate the nature of a change in the profile of circulating glucose; since endogenous glucose (^12^C) is influenced by production and disposal, whereas an orally administered tracer (^13^C‐glucose) is influenced by absorption and disposal, the total AUC can differ from the tracer‐based AUC [28]. When we probed metabolic activity using a ^13^C‐labeled glucose tolerance test, we did not observe any noticeable effect on the plasma AUC even though a lower blood glucose at baseline was observed (Figure 3Bi, inset left). Note that the AUC here uses the respective *t* = 0 min concentration as the baseline; we are examining the net AUC and not the total AUC. In this experiment, the glucose bolus contained [U‐^13^C_6_]glucose (1 g ∙ kg^−1^) which allowed a single study to multiplex readouts; MS analyses of the same plasma samples demonstrated that Semaglutide influenced the movement of orally administered ^13^C‐glucose (Figure 3Bi, inset right). Our example demonstrates how a tracer‐based readout can be impactful in the context of drug action; if we relied only on the change in total pool (net AUC) we would underestimate or miss the fact that drug was changing the metabolic (biochemical) trafficking. The mice were then utilized in a second study in which a bolus of ^2^H‐water was given with free access to food overnight. MS analyses of plasma‐derived analytes demonstrated reduced synthesis of a circulating triglyceride and apoA1 (Figure 3Bii); specific technical details can be found in the literature [17, 29]. These preliminary studies examined key downstream metabolic pathways involving carbon/nitrogen sinks; the effects do not necessarily derive from only changing glucose dynamics; Semaglutide likely redirects traffic flow throughout multiple biochemical networks. This pilot study did not aim to explain which roadway(s) were blocked; the main point is that stable isotope kinetic readouts can yield novel insight surrounding primary and secondary target‐mediated effects.

A final example shows the interplay of blocking cholesterol absorption and the potential for rebound in cholesterol synthesis (Figure 3C). Here, non‐human primates were studied during a baseline period and then randomized to Vehicle or Ezetimibe (Eze, 0.05 mg ∙ kg^−1^ ∙ day^−1^); treatment with Eze slightly reduced total cholesterol (Figure 3Ci). A triple tracer protocol [30] (^2^H_6_‐cholesterol, PO 5 mg ∙ kg^−1^; ^13^C_2_‐cholesterol, IV 5 mg ∙ kg^−1^; ^2^H‐water, PO 6 mL ∙ kg^−1^) demonstrated that Eze had a rather sizeable effect on the absorption pathway, but there was a compensation with respect to the contribution of de novo synthesis (Figures 3Cii and Ciii, respectively).

### 2.2 Utilizing Stable Isotope Tracing Methodology to Increase Quality Decision‐Making

Investing in MoA studies early in a program lifecycle can illuminate the biochemical kinetics in model systems, which may inform on target engagement and yield insight regarding biomarkers. We encourage researchers to build this foundational knowledge base during target validation and then immediately leverage it during screening; not only does this yield insight regarding pathway activity, but it enhances the translational mindset and bridges early discovery with clinical development. As we have demonstrated, the readouts highlight important interactions between biology and pharmacology.

**FIGURE 4:**
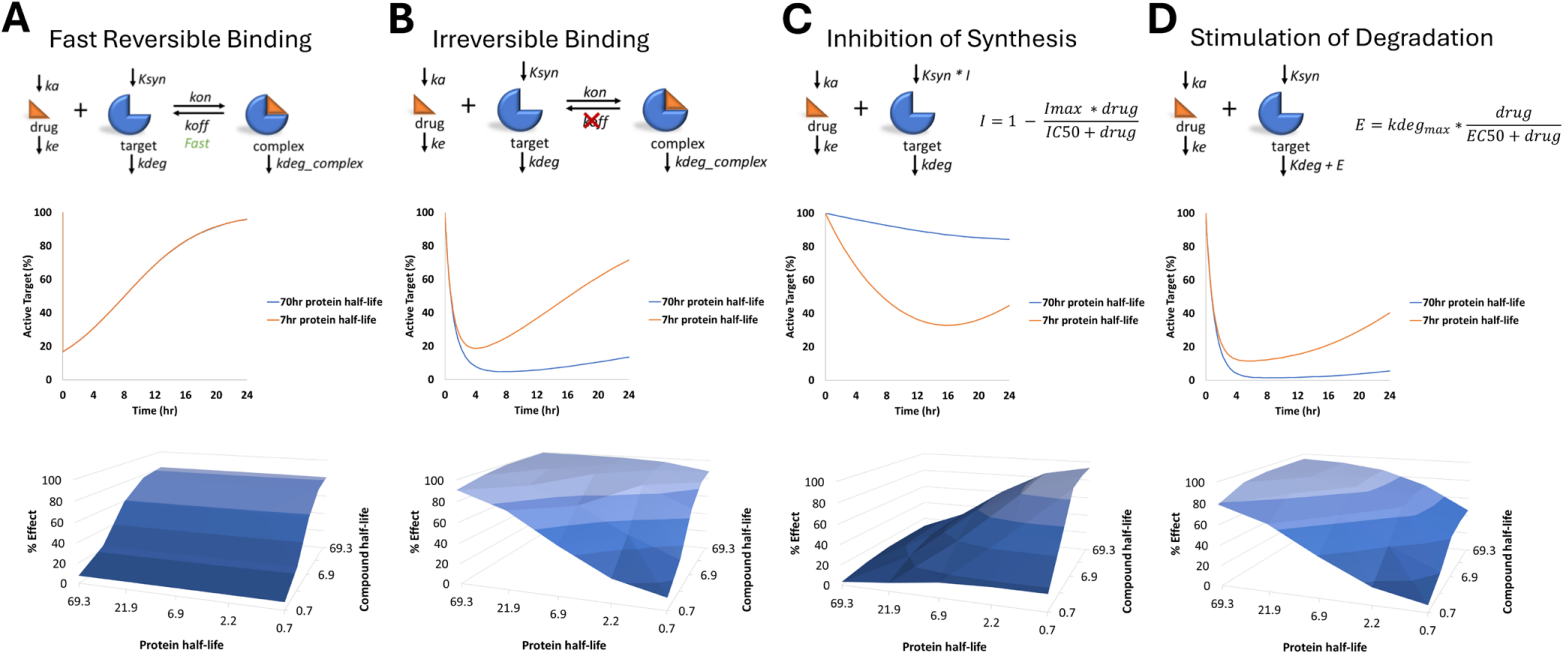
Protein kinetics in the context of modality selection and time course of response. The impact of protein (target) kinetics on the time course of response is dependent on the mechanism of action and binding interaction kinetics. Protein kinetics are typically not a factor when developing exposure‐response models in the context of rapidly reversible binding (Panel A). In contrast, protein kinetics become a critical factor when other modes of interaction are engaged (Panel B–D). In Panel A, target binding is rapidly reversible, while binding is irreversible in Panel B, yet they are both inhibiting active target due to target occupancy. In Panel C, active target is reduced due to inhibition of protein synthesis thus reducing target abundance, while reduced target abundance is achieved through stimulation of protein degradation in Panel D. Percent active target was simulated over time to demonstrate the impact of a short versus a long protein half‐life (7 or 70 h, respectively) on the time course of response, note that the same pharmacokinetic profile is assumed in the different scenarios (compound t_1/2_ ~ 3.5 h). In addition, the sensitivity of the overall effect (reduction of active target) to the compound half‐life vs. the protein half‐life was evaluated in each scenario, demonstrating the interplay between kinetics of compound elimination and protein turnover for different mechanisms of interaction. Note that protein kinetics are typically modeled by assuming zero‐order synthesis (Syn) and first‐order degradation (k_deg_), with steady state pool size defined by Syn/k_deg_.

Stable isotope methods can connect other aspects of pharmacokinetic–pharmacodynamic relationships and support different areas of development. The critical role of target turnover kinetics and its interplay with biochemical kinetics is discussed in a review by [31] and demonstrated with simulations in Figure 4. Knowledge of target protein turnover is used in guiding the compound dosing strategy and modeling the possible outcomes for compounds with differing interaction kinetics. For example, a relatively direct relationship is observed between pharmacokinetics and pharmacodynamics when rapidly reversible inhibitors are used to block enzyme activity (Figure 4A); the kinetics of compound elimination is the primary driver of response duration. However, when compounds exert their effects via non‐ (or very slowly) reversible covalent inhibition, the rate of target protein (re)synthesis becomes a prominent contributor to the duration of response (Figure 4B), and there is an immediate need to understand target protein kinetics. Understanding target half‐life can be informative when selecting a modality with the appropriate mechanism of action as well (Figure 4C,D); proteins with relatively short half‐lives can change more rapidly by limiting protein synthesis as compared to proteins with longer half‐lives (Figure 4C), while proteins with longer half‐lives benefit from a longer duration of response by stimulating degradation compared to proteins with short half‐lives (rapid turnover) (Figure 4D). An understanding of endogenous target protein kinetics is needed to best align pharmacokinetics and dosing intervals to achieve desired pharmacodynamics for a compound with a given mechanism of action and interaction kinetics.

## 3 Summary

The nature of the work discussed herein is multidisciplinary, requiring a collaborative combination of expertise in nutritional biochemistry, metabolic regulation, analytical chemistry, and mathematical modeling. Questions surrounding biochemical regulation are at the center of disease etiology and require answers to develop therapeutic interventions (Figure 1). This is especially important since drug invention can be limited by a lack of knowledge regarding underlying metabolic pathway activity, which can lead to efficacy‐based failures. In our experience, it can be pivotal to address these questions early during the drug invention process (Figure 2). Elucidating MoA when metabolic activity is being modulated yields knowledge about pathway connectivity, which can help rationalize chances for exerting maximal effects on endpoint concentrations; likewise, defining kinetics of marker pools can be useful in planning biomarker strategies (Figure 3). Finally, the kinetics of endogenous processes can have a direct impact when devising exposure‐response predictions and can inform on modality selection (Figure 4).

We have examined several studies taken directly from our collective experience. The examples discussed here emphasize when and how stable isotope kinetic methods can aid researchers to overcome common hurdles in the drug invention process. Troublingly, when programs backtrack or halt their screening plan upon discovering the types of issues highlighted here, they consume more resources across multiple functional areas than would be necessary if prudent studies were run early in a program's lifecycle. The importance of understanding biochemical kinetics in human physiology remains true whether one considers the typical forward translation of hits from a successful screen or reverse translation when trying to compare pathway tone that may be encountered in the clinic to the model used in screening [32, 33]. In many cases, the type of data we have discussed here can be generated as part of routine program support with minimal extra work. This is especially useful when thinking about relevant efficacy models to guide translation. Generating this knowledge before the team arrives at in vivo studies allows data‐informed strategic decisions based on a streamlined screening plan with a line of sight to clinical developability. Teams can help ensure delivery of high‐quality candidate molecules with the potential to impact disease biology and with integral information to implement biomarker strategies.

Our case studies have largely focused on the use of stable isotopes where drugs are being developed in the context of unknown or multifactorial disease biology and are not without caveats. For instance, in our previously mentioned DGAT2 example, it remains unclear clinically whether interventions based on DGAT2 enzymatic inhibition will result in the desired efficacy. However, it is important to recognize that directed modalities besides traditional small‐molecule chemistries may also similarly benefit from isotope tracing applications. Replacement or modulation of target functionality via organ transplantation, cell‐, or gene‐based therapies has made remarkable progress over the last few decades, and stable isotope tracer methods can continue to provide critical support in the emergence of this new wave of therapeutics [34]. For example, defects in any of the six enzymes or two transporters comprising the urea cycle (UCD) can cause severe inherited metabolic diseases, with deficiency in the enzyme ornithine transcarbamylase (OTC) estimated to be causal in over half of all patients with confirmed urea cycle disorders (UCD) [35]. Mouse models of UCD suggest that even a minor increase in hepatic activity of a deficient enzyme could significantly alleviate a severe UCD phenotype and reduce patient treatment burden [36, 37]. Indeed, investigators using a ^15^NH_4_Cl tracer bolus confirmed that two children with partial OTC deficiency achieved normal ureagenesis post‐liver transplantation [38]. Excitingly, a phase II clinical trial using lipid nanoparticle messenger RNA therapy (ARCT‐810 under clinicaltrials.gov ID NCT05526066) for OTCD is underway, and is utilizing a ^13^C‐urea tracer to quantify change from baseline in urea cycle function as a secondary trial outcome measure. Measures that help researchers and clinicians determine the magnitude of change in metabolic flow can be used to determine the effectiveness of restoring function to the metabolic pathway and guide treatment regimens. In the case of patients with methylmalonic acidemia (MMA), researchers found that a breath test utilizing [1‐^13^C]propionate as a measure of whole‐body propionate oxidative capacity was able to successfully predict both disease severity and treatment outcome, thus supporting the use of this tracer as a pharmacodynamic response biomarker in MMA [39].

Researchers seeking to navigate the path from target validation, screening, preclinical in vivo testing, and onto eventual human studies can be met with various unexpected outcomes at every step and, unfortunately, these types of scenarios are commonly encountered in drug discovery. Stable isotope‐based studies of biochemical pathways can be essential in successfully guiding program development forward or in making the critical but necessary decision to terminate a program. When used routinely, we see that teams often gain new knowledge regarding pathway connectivity, their associations with broader biochemical networks, and provide future utility across different areas of human health.

## 4 Nomenclature of Targets and Ligands

Key protein targets and ligands in this article are hyperlinked to corresponding entries in http://www.guidetopharmacology.org, the common portal for data from the IUPHAR/BPS Guide to PHARMACOLOGY and are permanently archived in the Concise Guide to PHARMACOLOGY 2023/2024 [40, 41].

## Author Contributions

R.A.K. and S.F.P. wrote the manuscript. S.F.P. designed the research. R.A.K., D.Z., B.L., H.Y., D.D., and R.V. performed the research. R.A.K., H.Y., J.E.B., and S.F.P. analyzed the data. D.M., H.K., and J.P. contributed ideation and support to the research. Animal studies were conducted using protocols that were approved by Merck and Co. Inc., Rahway, NJ, USA Institutional Animal Care and Use Committee.

## Conflicts of Interest

Current affiliations: D.M., Aditum Bio, Oakland, CA, USA; D.D., University of Texas Southwestern Medical Center, Dallas, TX, USA; R.V., Deep Apple Therapeutics, San Francisco, CA, USA; H.K., Pioneering Medicines, Cambridge, MA, USA; S.F.P., PharmaCadence Analytical Services LLC, Hatfield, PA, USA.

## Data Availability

The authors have nothing to report.
